# *In vivo* Magnetic Resonance Metabolic and Morphofunctional Fingerprints in Experimental Models of Human Ovarian Cancer

**DOI:** 10.3389/fonc.2016.00164

**Published:** 2016-06-28

**Authors:** Rossella Canese, Delia Mezzanzanica, Marina Bagnoli, Stefano Indraccolo, Silvana Canevari, Franca Podo, Egidio Iorio

**Affiliations:** ^1^Department of Cell Biology and Neurosciences, Istituto Superiore di Sanità, Rome, Italy; ^2^Department of Experimental Oncology and Molecular Medicine, Fondazione IRCCS Istituto Nazionale dei Tumori, Milan, Italy; ^3^Immunology and Molecular Oncology Unit, IOV – Istituto Oncologico Veneto – I.R.C.C.S, Padova, Italy

**Keywords:** MRI, magnetic resonance spectroscopy, DWI, ADC, epithelial ovarian cancer, target therapy, animal model

## Abstract

Epithelial ovarian cancer (EOC) is the gynecological malignancy with the highest death rate, characterized by frequent relapse and onset of drug resistance. Disease diagnosis and therapeutic follow-up could benefit from application of molecular imaging approaches, such as magnetic resonance imaging (MRI) and magnetic resonance spectroscopy (MRS), able to monitor metabolic and functional alterations and investigate the underlying molecular mechanisms. Here, we overview the quantitative alterations that occur during either orthotopic or subcutaneous growth of preclinical EOC models. A common feature of ^1^H MR spectra is the presence of a prominent peak due to total choline-containing metabolites (tCho), together with other metabolic alterations and MRI-detected morphofunctional patterns specific for different phenotypes. The tCho signal, already present at early stages of tumor growth, and changes of diffusion-weighted MRI parameters could serve as markers of malignancy and/or tumor response to therapy. The identification by MRS and MRI of biochemical and physiopathological fingerprints of EOC disease in preclinical models can represent a basis for further developments of non-invasive MR approaches in the clinical setting.

## Introduction

Epithelial ovarian cancer (EOC) is the fifth cause of cancer-related deaths among women (1), leading to about 140,000 deaths worldwide per year. Standard treatment for advanced-stage EOC is debulking surgery followed by platinum-based chemotherapy, with high response rates, but most of these patients will eventually relapse. Several drugs are available to treat recurrences; however, clinical responses remain short-lived and lead to only marginal improvements in survival of patients with platinum-resistant disease (2).

Prognostic and predictive biomarkers of therapy response need to be identified, and novel approaches of drug response assessment need to be developed to validate additional biological end points.

Early detection of response to cancer treatment frequently presents a challenge, as many new therapies lead to inhibition of tumor growth rather than to tumor shrinkage. Among the new therapeutic agents under clinical development, those with relative late effects could strongly benefit from an early indication of tumor response to treatment. Novel non-invasive methods to monitor or predict response to treatment, therefore, need to be developed.

Magnetic resonance spectroscopy (MRS) is a non-invasive imaging method that can be employed to monitor metabolism alterations that are associated with early drug target modulation (3) and can be predictive of cancer response to treatment (4). After controversial results in the differentiation of benign vs. malignant lesions in EOC patients, probably due to the heterogeneous nature of this disease (5), recent clinical studies are evaluating the role of the altered spectral pattern as an indicator of response during treatment of malignant disease rather than as initial diagnostic tool.

Magnetic resonance imaging (MRI) is the standard modality for the local staging of gynecological malignancies but has several limitations, particularly for lymph node staging and evaluation of peritoneal carcinomatosis. Recent growing interest is addressed to functional imaging modalities, such as diffusion-weighted MRI (DWI), a unique technique that provides tissue contrast by exploiting the restricted water mobility within hypercellular tumors to increase the contrast between these lesions and surrounding tissues. Using quantitative measurement of apparent diffusion coefficient (ADC) or other perfusion-related parameters, DWI provides a new tool for better distinguishing malignant tissues from benign tumors (6) and can aid in early monitoring of treatment efficacy in patients with advanced EOC (7). Moreover, the capability of DWI to detect alterations at cellular level rather than in the entire tumor mass could be crucial in the assessment of early response to a targeted therapy.

Important advances in our understanding of the molecular EOC pathogenesis and tissue heterogeneity were achieved by using appropriate preclinical models that approach the complexity of this disease in humans. Proper preclinical models that mimic tumor behavior and microenvironment involvement in patients are also essential for the evaluation of novel treatments (8, 9). Among these, models of spontaneous EOC in experimental animals (10, 11) or genetically modified animals (12, 13) have been developed, but their use remains limited because of the long latency to tumorigenesis and the heterogeneity in the timing of tumor development. EOC cell lines derived from ascites or primary ovarian tumors have been extensively used for studying tumor growth and response to treatment (14). Xenografts derived from intraperitoneal (i.p.) injection of human EOC cells in immunodeficient mice are relatively rapid to generate and develop tumor masses mimicking tumor diffusion in patients; together with subcutaneous (s.c) xenografts, these models have been widely used for studying the mechanisms controlling tumor growth and chemosensitivity. An alternative orthotopic EOC model can be generated by directly engrafting onto the ovary of female mice a piece of tumor tissue derived from an ovarian tumor xenograft (15).

In this short review, we will discuss the significance of metabolic and functional features, respectively, detected by MRS and DWI in different EOC preclinical models, focusing on aspects of metabolic reprogramming occurring in response to anticancer treatment strategies. In spite of the growing interest in the use of MRI (especially DWI) techniques in ovarian cancer clinical studies (16–18), a still limited number of papers have been published on *in vivo* experimental EOC models, mainly due to inherent experimental difficulties. For this reason, we decided to also include, in this review, some reports presented at prestigious peer reviewed international conferences (with abstracts published in copyrighted proceedings), although not yet translated into *in extenso* articles.

## Metabolic Features Detected by *In Vivo* ^1^H MRS in EOC Xenografts

**Figure 1:**
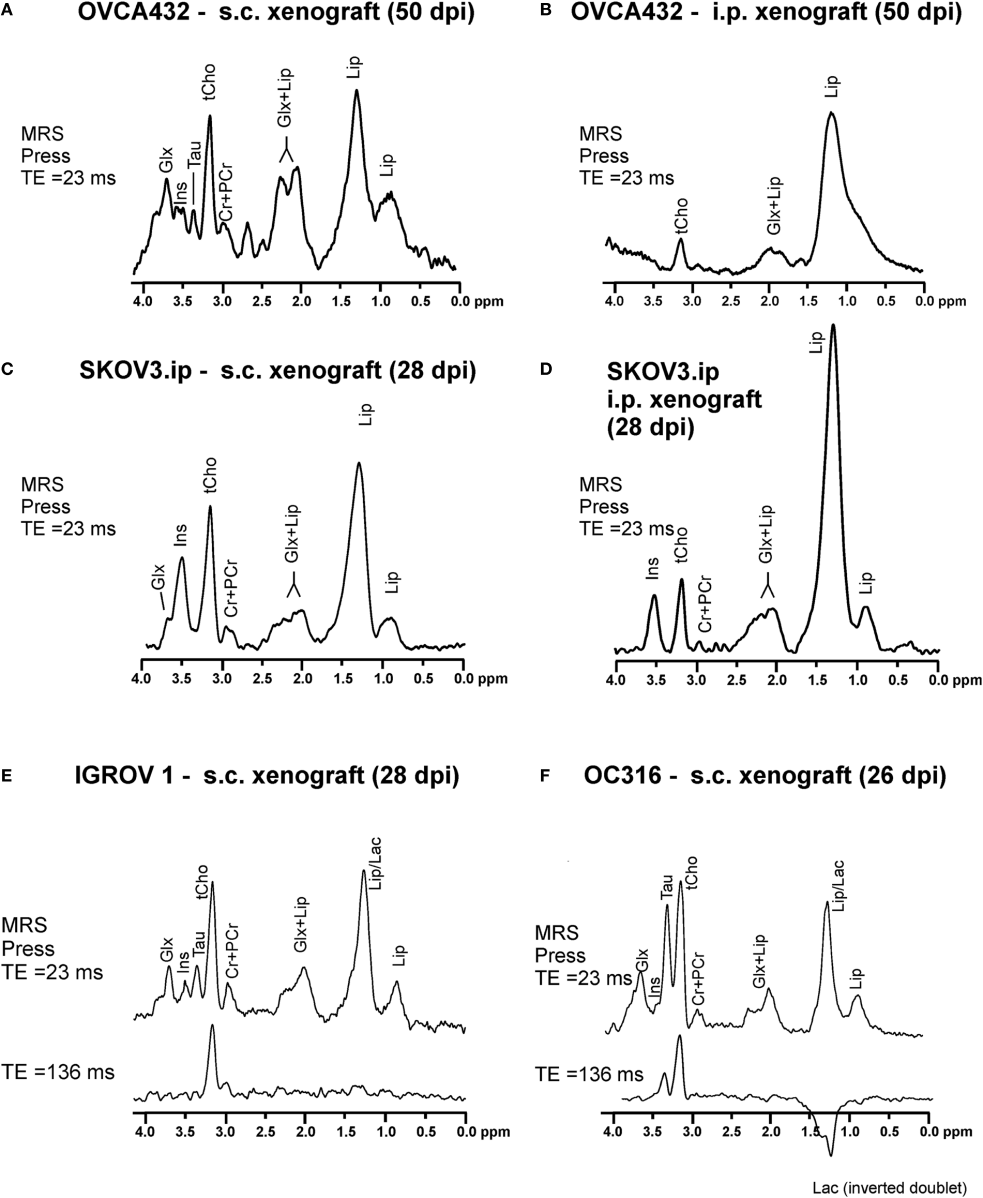
**Typical *in vivo*^1^H MRS spectra acquired at 4.7 T from vital areas (as detected by T2-weighted MRI) of xenografts of about 400–600 mm^3^ obtained in SCID mice by (A) subcutaneous (s.c.) implantation of OVCA432 cells (ATTC cell line derived from patient ascite), (B) intraperitoneal (i.p.) implantation of OVCA432 cells, (C) s.c. implantation of the SKOV3.ip cells [a cell line obtained by *in vivo* passage and subsequent *in vitro* culture of the HER2-positive ATTC SKOV3 cell line** (14)**], (D) i.p. implantation of SKOV3.ip cells, (E) s.c. implantation of IGROV1 cells (ATTC cell line derived from human primary ovarian cancer), and (F) s.c. implantation of OC316 cells (cell line derived from patient ascite)**. All these EOC models were examined between 26 and 50 days post implantation (dpi). Peak assignment: Glx, glutamine *plus* glutamate; Ins, myo-inositol; Tau, taurine; tCho, total choline-containing compounds; Cr + PCr, creatine *plus* phosphocreatine; Lip, lipids; Lac, lactate. Abbreviation: ppm, part per million. Further details in Ref. (22) for spectra in **(A,B)**, Ref. (21) for spectra in **(C,D)**, and Ref. (24) for spectra in **(E,F)**.

Typical ^1^H MR spectra acquired from vital areas of EOC models obtained by either s.c. or i.p. implantation of human EOC cell lines of different origins (19, 20) in immunocompromised mice (21, 22) are reported in Figures 1A–F. The choice of an s.c. implant relies on its simplicity and the short time (about 2 weeks) required to develop solid masses. The i.p. model, instead, recapitulates peritoneal spread, mimicking typical features of EOC growth and dissemination observed in patients. As shown in Figure 1, a prominent ^1^H MRS signal at 3.2 parts per million (ppm) arises from the trimethylammonium headgroups of the total choline-containing metabolites (tCho). This peak could be clearly detected and quantitated in all investigated EOC models at different times of tumor growth. A complete set of MRS analyses at different echo times and at different time points during tumor growth, together with the application of a quantitative method including measurements of water T2 and content (21), allowed quantification of the spectral changes occurring over time. The tCho level was found to range between 2 and 6 mM in all analyzed xenografts (Figures 1A–F).

According to high resolution ^1^H MRS analyses of cancer cell (23) and tissue extracts (21, 24), the tCho peak mainly comprises contributions from phosphocholine (PCho), glycerophosphocholine (GPCho), and free choline (Cho). These analyses showed that the PCho/tCho ratio in all cells used for implants shown in Figure 1 was greater than 0.70 (21, 24).

Besides tCho, multiple signals arising from the glutamine and glutamate pool (Glx), myo-inositol (Ins), and taurine (Tau) are also detected in the spectra of EOC xenografts (Figures 1A–D). Xenografts obtained from s.c. implantation of SKOV3.ip cells [a highly tumorigenic cell variant of the ATCC SKOV3 cell line, endowed with a twofold higher HER2 overexpression level (14)], showed that the mean tCho content ranged from 2.9 to 4.5 mM (with a PCho/tCho ratio of 0.90). In the SKOV3.ip s.c. model, the Glx signal was detected at early stages of tumor growth and decreased at later stages, while Ins increased in vital areas of the tumors (21). When the tumor reached a size larger than 400 mm^3^, an increase in lipid (Lip) signal at 1.3 ppm was also observed in “normal appearing” areas in which the presence of micronecrosis was detected. Spectral editing ensured that this signal was mainly due to lipids rather than to lactate (Lac) (21).

An increased Lip resonance was also observed in the viable part of i.p. implants, either OVCA432 (Figure 1B) or SKOV3.ip models (Figure 1D), compared with the corresponding s.c. implants (Figures 1A,C, respectively). This was due to the presence of round-shaped structures compatible with lipid aggregates detected by H&E staining in *ex vivo* analyses (21).

Peculiar spectral profiles have also been observed in s.c. xenografts derived from OC316 and IGROV1 cells (Figures 1E,F). These patient-derived EOC cell lines have been identified as prototypes of highly and poorly glycolytic EOC cells, respectively (25), based on measurements of glucose consumption and lactate production rates *in vitro*, as well as assessment of expression levels of glycolysis-associated genes. Besides the tCho and lipid signals, high resonances arising from Tau and Lac were detected in the spectra of “glucose addicted” EOC xenografts (OC316) – but not in those of the low glycolytic IGROV1 EOC model. The Lac resonance could be isolated from the partially overlapping Lip resonance in the *in vivo* ^1^H MR spectra of the OC316 model by exploiting the property of the Lac signal to be reversed when acquired with an echo time (TE) of 136 ms and the property of Lip signal to be decayed at TE = 136 ms due to its short T2 value, as shown in the lower spectrum of Figure 1F and confirmed by bioluminescence metabolic imaging (24).

Overall, this body of evidence showed that an elevated tCho peak was the most common feature of *in vivo* ^1^H MR spectra of the investigated EOC models, in agreement with similar findings reported for clinical EOC lesions (26–28). Moreover, thanks to the shorter echo times achievable in preclinical studies [about 20 ms vs. 135 ms typically used for clinical MRS and ^31^P MRS and spectroscopic imaging (MRSI) examinations], we could observe and quantify several other metabolites, which give a more detailed picture of tumor metabolism and may allow for a more extensive “metabolic targeting.”

## The tCho Peak as a Marker of Malignancy and Response to Therapy

The concentration levels of the tCho peak components depend on the activity rates of multiple enzymes involved in anabolic and catabolic pathways of the agonist-activated phosphatidylcholine cycle (29). Alterations in the levels of the tCho components in cancer may result from changes occurring in the activity rates of multiple enzymes of this cycle under the dysregulated control exerted by oncogene-driven cell signaling cascades (23, 30). An increase in the tCho content has been indicated as a biomarker for distinguishing malignant from benign lesions in the breast (31, 32) and has been detected in a variety of other cancer cells and tissues (23), including EOC cells (19, 20) and clinical lesions (19, 20, 33). In particular, a 4- to 7-fold increase in PCho detected in a series of human EOC cell lines was associated with a 12- to 25-fold activation of choline kinase (ChoK) and a 5- to 17-fold activation of phosphatidylcholine-specific phospholipase C (PC-PLC) (20).

A number of preclinical studies have been addressed to evaluate the role of tCho and its components (especially PCho) as possible markers of tumor progression and response to therapies in different types of cancers (23). Among these, recent investigations also focused on MRS-detected metabolic effects induced on EOC models by anticancer treatments.

Reduction of the tCho content of SKOV3.ip xenograft after cytotoxic treatments suggests that this signal could be a potential biomarker of treatment response. Decreased tCho levels and increased Lac content, associated with an increase in diffusion parameters (both diffusion and perfusion components), were found to be induced in a SKOV3.ip xenograft model by trabectedin (a new marine-derived antitumor agent, which has shown activity in multiple tumor types, including EOC). The detected tCho reduction suggests that this signal could be a potential biomarker of trabectedin response, while the Lac increase likely reflects the activation of lactic dehydrogenase (LDH) as a consequence of a cytotoxic damage to the cancer cells (34).

The effects exerted on tumor growth and metabolism by panthetine, a derivative of vitamin B5 and precursor of coenzyme A, were investigated by Penet et al. (35) using MRI/MRS in an orthotopic model of ovarian cancer obtained by engrafting a piece of OVCAR3 tumor tissue onto the ovary of SCID female mice. Pantethine treatment resulted in slower tumor progression, decreased levels of PCho and phosphatidylcholine, and reduced metastases and ascites occurrence.

A marked tCho reduction has also been observed *in vitro* (in SKOV3.ip cells) and *in vivo* (in SKOV3.ip s.c. xenografts) upon treatment with the competitive PC-PLC inhibitor tricyclodecan-9-yl-potassium xanthate (D609). The *in vivo* tCho reduction was associated with increases in the T2 and ADC mean values, along with reduced Ki67 index and HER2 content, suggesting that PC-PLC plays an important role in HER2-driven EOC cell signaling and tumorigenicity (36).

## pH Alterations in EOC Models and Its Potential Effects on Cancer Treatment

The tumor microenvironment plays a key role in tumor malignancy (37). In particular, microenvironment acidity has been shown to have a role in the resistance to chemotherapy, proliferation, and tumor progression. The causes of acidic extracellular pH in tumors have not yet been fully elucidated, although important contributions probably arise from deficiencies in blood perfusion, metabolic abnormalities associated with transformation, and an increased capability for transmembrane pH regulation (23).

^31^P MRS and spectroscopic imaging offers the most powerful approaches currently available to non-invasively measure extracellular pH (pHe) and intracellular/extracellular pH gradient (ΔpH) in intact cancer cells and tissues (38, 39). In fact, by measuring the chemical shift difference between the exogenous cell-impermeant ^31^P reporter 3-aminopropyl phosphonate resonance (3-APP, administered i.p. immediately prior to the MRI/MRS analyses) and that of α-ATP, the acidic pHe (6.7–6.8) for s.c. and i.p. SKOV3.ip models has been measured at 30–35 dpi. The intracellular pH (pHi) measured from the chemical shift difference between Pi and α-ATP was 7.3 in s.c. and 7.1 in i.p. xenografts (21).

An emerging technique, acidoCEST-MRI, utilizes iopromide, a contrast agent that contains two chemical exchange saturation transfer (CEST) effects and can assess *in vivo* pHe more accurately and with a higher spatial resolution than ^31^P MRS (40). AcidoCEST-MRI was applied to the orthotopic (i.p.) SKOV3 model to investigate the relationship between pHe, vascular perfusion, and tumor volume (40). This tumor model was mildly acidic, with an average pH of 6.88, in agreement with our findings. Additionally, larger tumors were found to be more acidic. A lower vascular perfusion allowed for an elevated lactic acid production, thus causing an increase in tumor acidosis.

These pH alterations observed in EOC models can reflect microenvironment alteration in EOC patients’ cancer lesions and may modulate their response to conventional or targeted therapies. The negative pH gradient detected in EOC models impairs the uptake of weakly basic drugs but can facilitate a selective retention by tumor tissues of drugs behaving as weak acids. MR techniques provide unique tools to selectively investigate these aspects *in vivo*.

## Differences in Cytotoxic and Cytostatic Drugs’ Effects as Detected by *In Vivo* DWI and MRS

Early monitoring of treatment efficacy in patients with advanced EOC can be achieved using DWI and ADC mean values and distributions. In particular, the shape of the ADC distribution (in terms of skewness and kurtosis) has been proven to reflect chemotherapy response in patients (7, 41), as well as in EOC models (14, 24).

By analyzing ADC mean values and distributions, differences in cytotoxic and cytostatic effect can be observed largely before tumor shrinkage. In fact, cytostatic treatment effects can be observed during or up to 48 h after the end of treatment due to cell swelling, which results in an ADC decrease during the treatment, in absence of any detectable alteration in tCho level. Although cell swelling is a phenomenon that occurs during all treatments (it is in fact the first manifestation of almost all forms of injury to cells), it is dominated by cell death when a cytotoxic agent is administered resulting in an increased extracellular space, which can be observed as an increased ADC, associated with a tCho reduction. Notably, the effect of a cisplatinum-based treatment on the SKOV3.ip model (s.c. implant) was to reduce the ADC mean value (14). On the same EOC model, cytotoxic agents, such as D609 or trabectedin, induced a marked mean ADC increase associated with tCho reduction (34, 36).

## EOC Models with Different Glycolytic Phenotypes have Different Responses to a Human VEGF Neutralizing mAB as Detected by *In Vivo* MRS and DWI

Xenografts obtained from OC316 and IGROV1 patient-derived EOC cells (described in paragraph 2) were used to investigate the effects on glucose metabolism of A4.6.1, a human VEGF neutralizing mAB bearing the same CDR region as bevacizumab (24).

In the “glucose addicted” OC316 xenografts, A4.6.1 caused a dramatic depletion of glucose and an exhaustion of ATP levels in tumors associated with the presence of large necrotic areas and partial tumor regression. Different behavior was observed in the IGROV1 xenograft where metabolism remained unaltered after anti-VEGF treatment associated with a minor effect on tumor growth. Functional links between AMPK pathway and therapeutic responses to VEGF neutralization have also been detected in EOC models.

**Figure 2:**
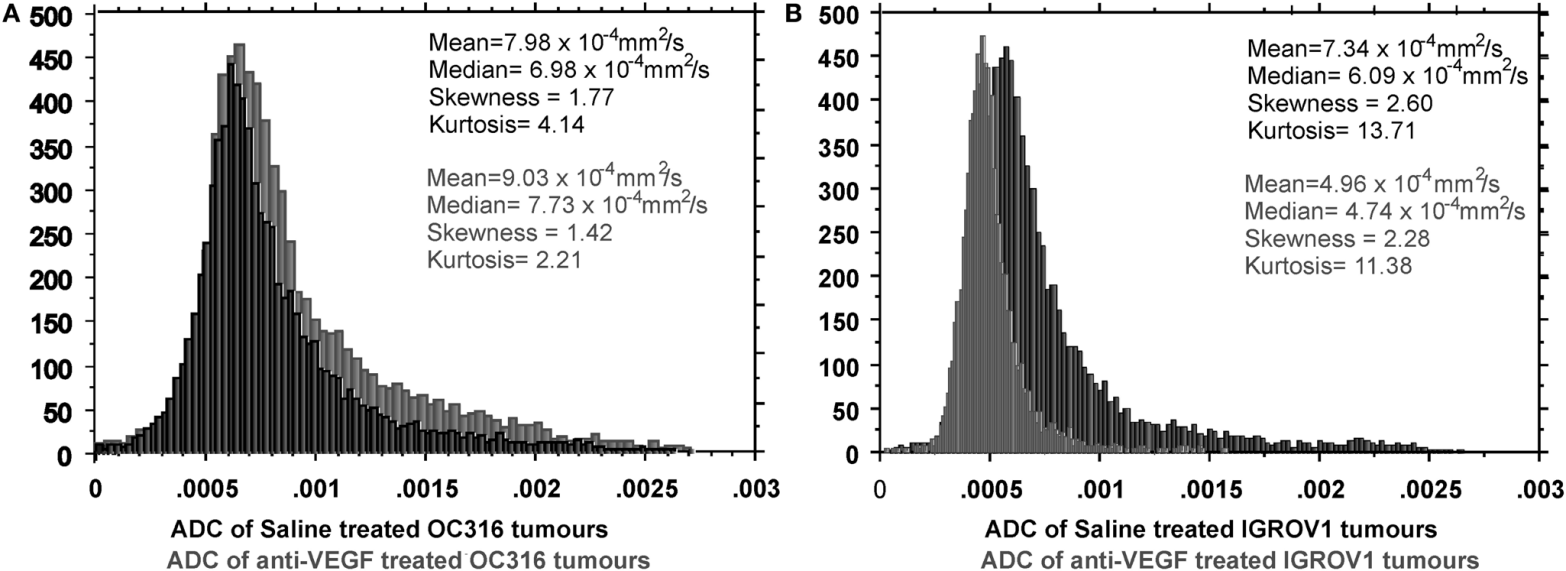
**Differential response to VEGF neutralization of two EOC models (OC316 and IGROV1) characterized by *in vitro* different glucose consumption and lactate production rates**. In the “glucose addicted” model, **(A)** an average increase of ADC is present corresponding to increased necrotic areas. In the IGROV1 model, **(B)** the A4.6.1 mAB induces a cell damage that can be observed as “cell swelling,” which causes the ADC reduction in the mean value and the right-hand shift of the ADC peak. Further details in Ref. (24).

Different ADC distributions characterized the response to VEGF neutralization of those two EOC models. In fact, as early as after 1 week of treatment, in the “glucose addicted” OC316 model, a cytotoxic effect of the anti-VEGF treatment could be observed as increased mean ADC (Figure 2A). This effect could be attributed to an increase in the extracellular space in agreement with microscopic or macroscopic areas of necrosis detected by histology (24) and associated with a significant delay in tumor growth. Opposite effect (ADC reduction) can be detected in the IGROV1 xenografts, where anti-VEGF treatment probably induces a cytostatic effect associated with a minor delay in tumor growth (Figure 2B). Moreover, the perfusion component [in terms of vascular signal fraction (VSF)], i.e., the component of fast diffusing spins (42), is reduced after anti-VEGF therapy, more pronounced in the OC316 than in the IGROV1 xenografts.

Prospectively, *in vivo* lactate and ADC quantitation and their monitoring following anti-VEGF treatment by MRI/MRS could represent non-invasive tools for the identification of “glucose addicted” EOC tumors and for predicting their clinical responses to bevacizumab and/or other antiangiogenic drugs.

## Future Prospectives with Radiolabeled Compounds for Positron Emission Tomography

Positron emission tomography (PET) imaging is mainly used to visualize general tumor processes, such as glucose metabolism using 18F-fluorodeoxyglucose (18F-FDG) and DNA synthesis using 18F-fluorodeoxythymidine (18F-FLT), as tools to predict prognosis and response to therapies (43, 44). More specific labeled ligands have been evaluated for specific targets, including immunotherapy components (64Cu-labeled anti-CA125 monoclonal antibody in SKOV3 and OVCAR3 xenografts) (45) and cell surface receptors (HER2 expression in SKOV3 xenografts) (46).

Despite the success in other cancers, the use of ^11^C-Cho-PET has been rarely applied to EOC with limited results (47), and to the best of our knowledge, there are no papers on preclinical models. Deuterium-substituted 18F-fluoromethyl-[1,2-^2^H_4_]-choline is a recently developed stable radiotracer that overcomes the short physical half-life of ^11^C and seems to be a promising tool for choline metabolism imaging for tumors with high PCho level such as ovarian cancer (48).

## Conclusion on the Usefulness of MRS and DWI in the Management of EOC Models

The use of multidisciplinary approaches including MRI and MRS in suitable EOC preclinical models may enhance understanding of molecular mechanisms of disease progression and response to therapy, eventually leading to the design of improved treatment strategies.

## Author Contributions

The manuscript was written by RC; revised by EI, FP, SC, DM, SI, and RC. All co-authors have been directly involved in obtaining part of the results described in the manuscript and read and approved it.

## Conflict of Interest Statement

The authors declare that the research was conducted in the absence of any commercial or financial relationships that could be construed as a potential conflict of interest.
